# About Thumbs

**Published:** 1874-03

**Authors:** 


### ABOUT THUMBS.

WE suppose that all our readers know that man would not be what he is without the thumb. This little fact has been so impressed upon us from our school days that we are not likely to forget it. Without the thumb for a lever we would be unable to hold anything tightly, and most of the inventions of our era would be useless, not to speak of the enormous general power that would be lost. Let us accept the fact of having thumbs, then, and be thankful, and rejoice over our Darwinian friends, the apes. We did not know, however, until we saw it in print yesterday, that the thumb represented intelligence and affection. Born idiots frequently come into the world without thumbs. Infants, until they arrive at an age when intellect dawns, constantly keep their fingers folded above their thumbs, but they soon know better, and, as the mind develops, recognize the dignity and usefulness of the despised digit. At the approach of death the thumbs of the dying, as if impelled by some vague fear, seek refuge under the fingers, and when thus found are an almost certain announcement of the end. So, in leaving this world, it would seem that our hands, in their last desire for movement, assume, with our growing unconsciousness, the same suggestive position in which the hands of the new born babe, with faculties all dormant, first shape themselves. Small thumbs denote an affectionate disposition; long thumbs go with long heads; short, thick, stumpy thumbs mark a cruel man, and much more is told us of the same kind.—*Balt. Gazette.*

